# Long Non-coding RNA lnc-GNAT1-1 Suppresses Liver Cancer Progression via Modulation of Epithelial–Mesenchymal Transition

**DOI:** 10.3389/fgene.2020.01029

**Published:** 2020-09-24

**Authors:** Jianchu Wang, Wei Wang, Qianli Tang, Libai Lu, Zongjiang Luo, Wenchuan Li, Yuan Lu, Jian Pu

**Affiliations:** ^1^Department of Hepatobiliary Surgery, Affiliated Hospital of Youjiang Medical University for Nationalities, Baise, China; ^2^Clinic Medicine Research Center of Hepatobiliary Diseases, Affiliated Hospital of Youjiang Medical University for Nationalities, Baise, China

**Keywords:** long non-coding RNA, liver cancer, lnc-GNAT1-1, anti-tumour, EMT

## Abstract

Recent studies have investigated the modulatory roles of long non-coding RNAs in the onset and progression of liver cancer. The present study aimed to elucidate the role of lnc-GNAT1-1 in liver cancer development and to explore the underlying mechanisms. Quantitative real-time polymerase chain reaction was performed to measure the expression levels of lnc-GNAT1-1 in cancerous tissues from patients with liver cancer and in liver cancer cell lines. The proliferative ability and apoptotic rates of liver cancer cells were measured using the counting kit-8 (CCK-8), colony formation, and flow cytometry assays. The abilities to invade and migrate were measured using Transwell assays. Epithelial–mesenchymal transition (EMT)-related proteins, E-cadherin, N-cadherin, and vimentin, were measured using western blotting. A nude mouse model was injected with xenografts to evaluate tumor growth *in vivo*. Downregulation of lnc-GNAT1-1 was observed in cancerous tissues from patients with liver cancer and in liver cancer cell lines, and low expression levels of lnc-GNAT1-1 were related to advanced TNM stage. Lnc-GNAT1-1 knockdown promoted invasion, migration, and proliferation of liver cancer cells and inhibited apoptosis, while lnc-GNAT1-1 upregulation exerted the opposite effects. The expression levels of lnc-GNAT1-1 negatively correlated with *in vivo* tumor growth in a xenograft nude mouse model. Mechanistic experiments revealed that lnc-GNAT1-1 exerted anti-tumor effects in liver cancer cells by inhibiting EMT. In conclusion, this study suggests that lnc-GNAT1-1 suppresses liver cancer progression by modulating EMT.

## Introduction

Liver cancer was the fourth leading cause of cancer death in 2018 (Bray et al., 2018). It was also one of the five leading causes of death in China (Zhou et al., 2019). In recent years, tremendous advances have been made in liver cancer treatment, including liver resection, radiofrequency and microwave ablation, liver transplantation, and chemotherapy. The early detection rate of human cancer has increased, and this has improved survival (Couri and Pillai, 2019). Nevertheless, liver cancer detected at advanced stages remains difficult to treat and survival rates remain low. It is therefore essential to explore the molecular mechanisms underlying liver cancer initiation and progression to offer novel insights for treatment.

Long non-coding RNAs (lncRNAs), that is, those containing more than 200 nucleotides, are a type of non-coding RNA that regulate the initiation and development of various cancers (Cho et al., 2018; Kim et al., 2018; Tang et al., 2019). Several studies have reported that numerous functional lncRNAs play vital roles in the development of various cancers. For example, increasing the expression of lncRNA-PVT1 was reported to decrease the sensitivity of radiotherapy in lung cancer by targeting miR-195 (Wu et al., 2017). Increased lnc-ROR was detected in breast cancer tissues and overexpressed lnc-ROR promoted epithelial–mesenchymal transition (EMT) and tamoxifen resistance by acting as a molecular sponge for miR-205 and increasing the expression of ZEB1 and ZEB2, given the binding sites between miR-205 and ZEB2 (Zhang et al., 2017). Additionally, lnc-ROR also promoted estrogen independence and tamoxifen resistance by activating the MAPK/ERK signaling pathway (Peng et al., 2017). LncRNA-MALAT1 increased levels of β-catenin in nuclear and activated the Wnt/β-catenin signaling pathway to exert oncogenic effects in colorectal cancer (Ji et al., 2013). The important functions of lncRNAs in liver cancer progression were also reported by several studies. For example, Mo et al. (2019) reported that lncRNA-FAM99B affected the prognosis of hepatocellular carcinoma (HCC) and proposed that the molecule may serve as a novel therapeutic target for HCC. Increasing the expression level of lncRNA-PDPK2P promoted invasion, migration, and proliferation of HCC cells by modulating the PDK1/Akt/caspase 3 cascade (Pan et al., 2019). By activating the BMP pathway, lncRNA-HAND2-AS1 enhanced liver cancer stem cell self-renewal and initiated liver cancer progression (Wang et al., 2019).

Previous studies have indicated that lnc-GNAT1-1 plays an anti-tumor role in malignancies of the digestive tract, including colorectal (Ye et al., 2016) and gastric cancers (Liu et al., 2018). Nevertheless, there are no studies of lnc-GNAT1-1 in liver cancer. Therefore, in the present study, we measured the expression levels and explored the potential functions of lnc-GNAT1-1 in liver cancer. We found downregulation of lnc-GNAT1-1 in both liver cancer tissues and cell lines. Cancerous tissues with advanced TNM stage showed lower lnc-GNAT1-1 expression levels. Functional assays showed that lnc-GNAT1-1 suppressed liver cancer cell proliferation, invasion, and migration and promoted apoptosis. *In vivo* studies showed that increasing the expression levels of lnc-GNAT1-1 inhibited the growth of xenograft tumors and that lnc-GNAT1-1 knockdown contributed to cancer development. We also found that lnc-GNAT1-1 exerted anti-tumor effects and may be a new effective therapeutic target for liver cancer.

## Materials and Methods

### Clinical Sample Collection and Processing

A total of 42 paired normal–cancer liver tissues were obtained during surgery from the same patients. The target specimens were washed in DPEC water, cut using special scissors, and immediately stored in liquid nitrogen until RNA extraction. The diagnosis of liver cancer was confirmed through postoperative pathology and all patients were staged on the basis of the World Health Organization criteria for the classification of liver cancer. Before collecting these specimens, no patient had received anti-tumor therapy. Consent was obtained from all patients. We received approval from the ethics committee of the Affiliated Hospital of Youjiang Medical University for Nationalities, Baise, Guangxi Zhuang Autonomous Region, China.

### Cell Culture and Mice

The normal human liver cell line LO2 and cancerous cell lines QGY-7703, SMMC-7721, Huh7, Hep3B, and GSG7701 were purchased from the Shanghai Cell Bank. Cells were grown in Dulbecco’s modified essential medium containing 10% fetal bovine serum (FBS) and 100 μg/ml penicillin/streptomycin. We used a special incubator containing 5% CO_2_ and cultured target cells at 37°C. Female BALB/C nude mice (28–35 days old) were provided by the Animal Core Facility (Shanghai, China) and housed at 25°C under pathogen-free conditions.

### RNA Extraction and Quantitative Real-Time Polymerase Chain Reaction (qRT-PCR)

Total RNA was extracted from cancerous tissues of patients with liver cancer and from liver cancer cell lines using TRIzol reagent (Invitrogen, United States), following the manufacturer’s protocol. qRT-PCR was performed using SYBR II Premix Taq (Takara, Japan) according to the manufacturer’s protocol. GAPDH mRNA was used as the internal reference. All experiments were carried out in triplicate. The PCR primers used were designed as follows: lnc-GNAT1-1 forward: 5′-ATGTGTCCCCAGGTTCCTGTT-3′; lnc-GNAT1-1 reverse: 5′-CCCCTGAGGACTTGAGTAGC-3′; GAPDH forward: 5′-CTGGGCTACACTGAGCACC-3′; GAPDH reverse: 5′- AAGTGGTCGTTGAGGGCAATG-3′.

### Plasmid Construction and Transfection

The lnc-GNAT1-1 expression vector LV-lnc-GNAT1-1 was provided by Genepharm (Hangzhou, China). The sh-RNA used to silence lnc-GNAT1-1 in liver cancer cell lines and corresponding sh-NC were also purchased from Genepharm. The transfection was carried out using Lipofectamine 2000 (Invitrogen, United States) according to the manufacturer’s protocol. The QGY-7703 cell line was transfected with LV-lnc-GNAT1-1 to construct cell lines overexpressing lnc-GNAT1-1 and was screened with 2.0 μg/ml puromycin for 14 days. Sh-RNA was transfected into the SMMC-7721 cell line to construct cell lines silencing lnc-GNAT1-1 and these cells were also screened with puromycin (2.0 μg/ml) for 14 days. Fluorescence was measured after 48 h.

### Counting Kit-8 (CCK-8) Assay

After transfection, SMMC-7721 and QGY-7703 cells were seeded into 96-well plates containing approximately 1,000 cells per well. Then, we added 10 μl CCK-8 reagent to each well and used a microplate reader (Tecan, Mechelen, Belgium) to measure the optical density at 0, 24, 48, 72, and 96 h. This experiment was carried out three times.

### Colony Formation Assay

The transfected SMMC-7721 and QGY-7703 cells were seeded into six-well plates at 500 cells per well and were cultured for 7 days. After visible colonies were formed, 2 ml of 4% paraformaldehyde was added to each well for 15 min. After washing with phosphate-buffered saline (PBS), the wells were stained with a Giemsa stain kit for 30 min. The colonies were counted using an ordinary optical microscope.

### Flow Cytometry

The apoptotic rates of target cells were measured using an FITC/Annexin V Apoptosis Detection Kit (BD Biosciences, Franklin Lakes, NJ, United States) on the basis of the manufacturer’s protocol. SMMC-7721 cells transfected with sh-lnc-GNAT1-1 or LV-lnc-GNAT1-1 were seeded into six-well plates with 4 × 10^5^ cells per well for 24 h and then harvested and washed twice with PBS. Subsequently, the cells were suspended in 400 μl binding buffer and we added 5 μl propidium iodide and Annexin V-FITC into the cell suspension. Cells were incubated in the dark for 15 min at 37°C. Then, flow cytometry was used to measure the apoptotic rates of these transfected cells.

### Cell Migration and Invasion Assays

The invasion and migration assays of SMMC-7721 cells transfected with sh-lnc-GNAT1-1 and QGY-7703 cells transfected with LV-lnc-GNAT1-1 were performed using Transwell chambers with 8 μmol/l pore size (Corning, NY, United States). The target cells were seeded into the upper chamber at 1 × 10^5^ cells per well and cultured with serum-free medium to carry out the invasion (with Matrigel; Beijing, China) and migration assays. Medium containing 20% FBS was added into the lower chamber to serve as a chemoattractant. Using cotton swabs, we removed the non-invading cells and stained invasive or migrated cells with hematoxylin–eosin in the membranes after 24 h incubation.

### Subcutaneous Metastasis Model in Nude Mice

Twenty-four mice were randomly divided into four groups. Sh-NC- or sh-lnc-GNAT-1-transfected SMMC-7721 cells and LV-NC- or LV-lnc-GNAT1-1-transfected QGY-7703 cells were seeded into the subcutaneous area of the necks of the nude mice. The tumors were dissected from the mice and the tumor volumes were measured from 12 to 30 days at 3 day intervals. Tumor volume was calculated as (width × length × height)/2. Approval for all animal experiments was obtained from the Institutional Committee for Animal Research.

### Western Blotting

Cell suspension was added into 60 mm dishes at 1 × 10^6^ cells per well. Cells were collected and washed with PBS three times. Using RIPA lysis buffer (KeyGen BioTech, Nanjing, China), total protein was extracted from target cells according to the manufacturer’s protocol, and the protein concentration was measured using a bicinchoninic acid protein assay kit (KeyGen BioTech). Subsequently, 30 μg of protein was separated using 12% sodium dodecyl sulfate–polyacrylamide gel electrophoresis at 120 V for 1.5 h. Then, the proteins were transferred onto polyvinylidene difluoride membranes (Millipore, Billerica, MA, United States) at 300 mA for 1 h. After blocking the membranes with 5% non-fat skimmed milk for 1.5 h at 20°C, the membranes were incubated with the primary antibodies for 12 h at 4°C. Primary antibodies against GAPDH, N-cadherin, vimentin, and E-cadherin were purchased from Cell Signaling Technology (Denver, CO, United States). The next day, the membranes were further incubated with anti-mouse or anti-rabbit secondary antibodies for 1 h at 20°C. Following three washes with TBS-T (10 min per time), expression levels of these proteins were measured using an enhanced chemiluminescence detection kit (Thermo Fisher, United States).

### Statistical Analysis

All data were analyzed using SPSS 19.0 software (IBM, Chicago, IL, United States) and are expressed as means ± standard deviation. The paired Student’s *t*-test was used to analyze significant differences between groups. Significant differences are shown as *P* < 0.05.

## Results

### Downregulation of lnc-GNAT1-1 Was Detected in Liver Cancer Tissues and Cell Lines

**FIGURE 1:**
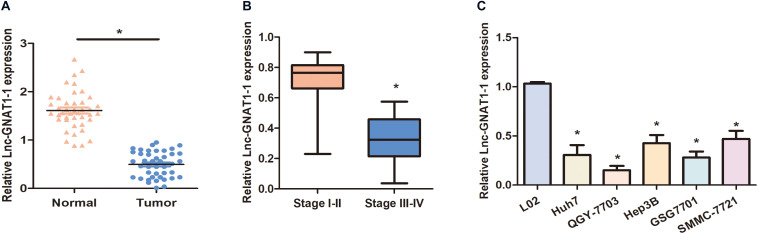
Downregulation of lnc-GNAT1-1 was observed in liver cancer tissues and cell lines. **(A)** Expression levels of lnc-GNAT1-1 in paired normal–cancer liver tissues. **(B)** Expression levels of lnc-GNAT1-1 in liver cancer tissues with various TNM stages. **(C)** Relative lnc-GNAT1-1 expression in liver cancer cell lines. *Significant differences compared with control groups are shown at *P* < 0.05. Each experiment was carried out in triplicate.

We carried out qRT-PCR to measure the expression levels of lnc-GNAT1-1 in 42 paired normal–cancer liver tissues. Significant downregulation of lnc-GNAT1-1 was observed in liver cancer tissues (Figure 1A). Low lnc-GNAT1-1 levels in liver cancer tissues negatively correlated with advanced TNM stage (Figure 1B). Downregulation of lnc-GNAT1-1 was also observed in the liver cancer cell lines (Huh7, QGY-7703, Hep3B, GSG7701, SMMC-7721) when compared with the normal liver cell line (LO2; Figure 1C). These findings suggest that lnc-GNAT1-1 correlated with the progression of liver cancer.

### Silencing lnc-GNAT1-1 Promoted Proliferation, Invasion, and Migration and Inhibited Apoptosis of the SMMC-7721 Liver Cancer Cell Line

**FIGURE 2:**
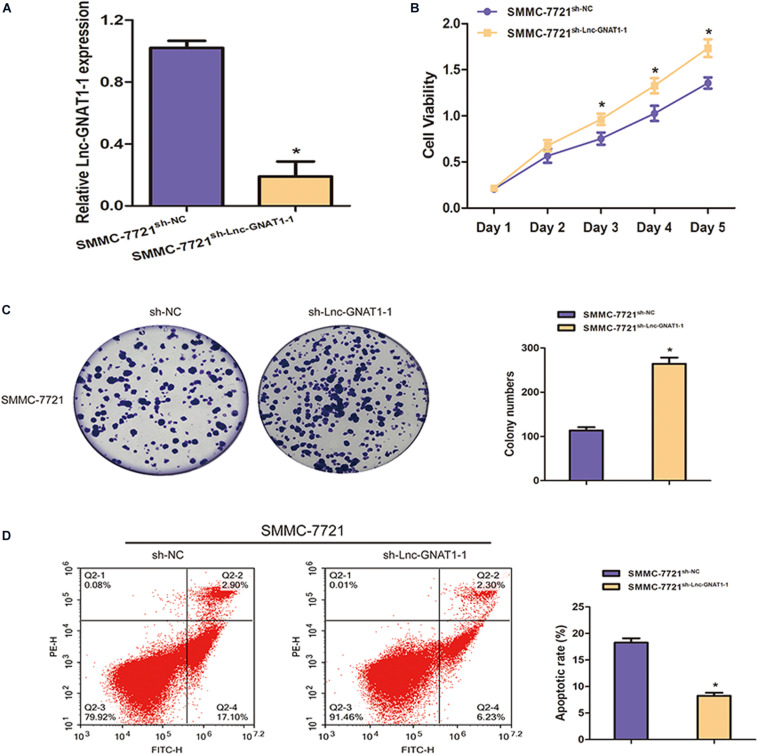
Lnc-GNAT1-1 knockdown enhanced cell proliferative ability and inhibited apoptosis in SMMC-7721 liver cancer cells. **(A)** The expression levels of lnc-GNAT1-1 in SMMC-7721 cells after transfection with sh-lnc-GNAT1-1 or sh-NC. **(B,C)** Cell proliferative ability of SMMC-7721 cells after sh-lnc-GNAT1-1 or sh-NC transfection was examined by CCK-8 assay **(B)** and colony formation assay **(C)**. **(D)** Cell apoptosis was evaluated by flow cytometry. *Significant differences are shown as *P* < 0.05. Each experiment was carried out in triplicate.

**FIGURE 3:**
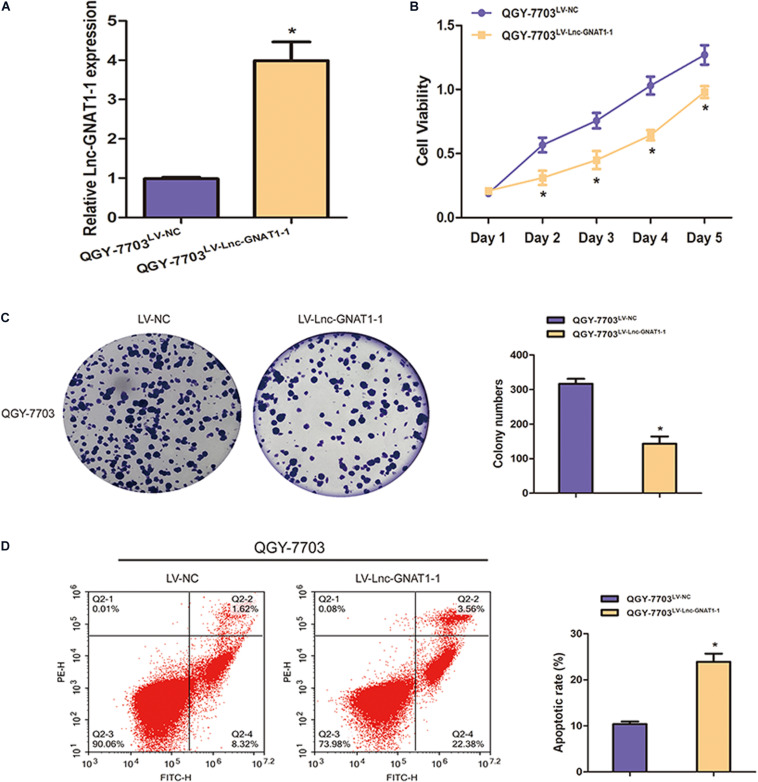
Lnc-GNAT1-1 silencing promoted the invasion and migration of SMMC-7721 liver cancer cells, while lnc-GNAT1-1 overexpression exerted an opposite effect in QGY-7703 cells. **(A,B)** Transwell assays were carried out to evaluate the ability to invade and migrate of SMMC-7721 cells after sh-lnc-GNAT1-1 or sh-NC transfection. **(C,D)** Transwell assays were carried out to evaluate the ability to invade and migrate of QGY-7703 cells after LV-lnc-GNAT1-1 or LV-NC transfection. *Significant differences are shown as *P* < 0.05. Each experiment was carried out in triplicate.

We constructed an lnc-GNAT1-1-silencing vector (sh-lnc-GNAT1-1) and used an empty vector for comparison. Expression levels of lnc-GNAT1-1 in the control group were approximately fivefold higher than those in the silencing group (Figure 2A). The CCK-8 assay indicated that the proliferative ability of SMMC-7721 cells transfected with sh-lnc-GNAT1-1 was greater than that of cells in the sh-NC group, and this result was consistent with that of the colony formation assay (Figures 2B,C). Cell invasion and migration assays revealed that the ability to invade and migrate was significantly enhanced in the sh-lnc-GNAT1-1 group (Figures 3A,B). Apoptosis was suppressed after sh-lnc-GNAT1-1 transfection in SMMC-7721 cells (Figure 2D).

### Upregulation of lnc-GNAT1-1-Inhibited Liver Cancer Cell Proliferation, Invasion, and Migration and Increased Apoptosis Rates

**FIGURE 4:**
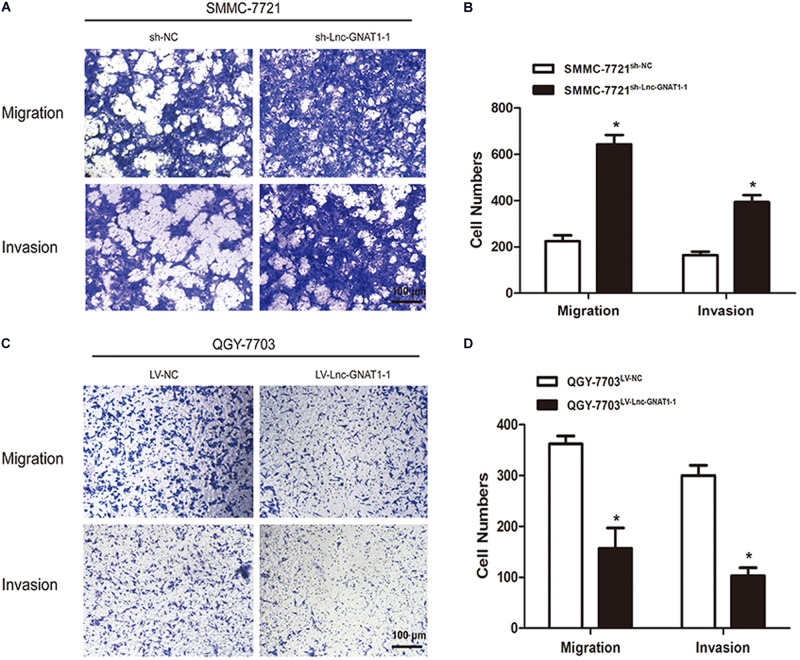
Lnc-GNAT1-1 overexpression suppressed cell proliferation and promoted apoptosis in QGY-7703 liver cancer cells. **(A)** Quantitative real-time polymerase chain reaction analysis of lnc-GNAT1-1 expression levels in QGY-7703 cells after transfection with LV-lnc-GNAT1-1 or LV-NC. **(B,C)** Cell proliferative ability of QGY-7703 cells after LV-lnc-GNAT1-1 or LV-NC transfection was detected by CCK-8 assay **(B)** and colony formation assay **(C)**. **(D)** Apoptosis was evaluated by flow cytometry. *Significant differences are shown as *P* < 0.05. Each experiment was carried out in triplicate.

LV-lnc-GNAT1-1 was transfected into the QGY-7703 liver cancer cell line to upregulate the expression of lnc-GNAT1-1 (Figure 4A). The proliferation, invasion, and migration of QGY-7703 cells were significantly inhibited by overexpression of lnc-GNAT1-1 compared with values from the control group (Figures 3C,D, 4B,C). Apoptosis was markedly induced after LV-lnc-GNAT1-1 transfection (Figure 4D).

### Lnc-GNAT1-1 Suppressed *in vivo* Liver Cancer Growth of Xenograft Tumors

**FIGURE 5:**
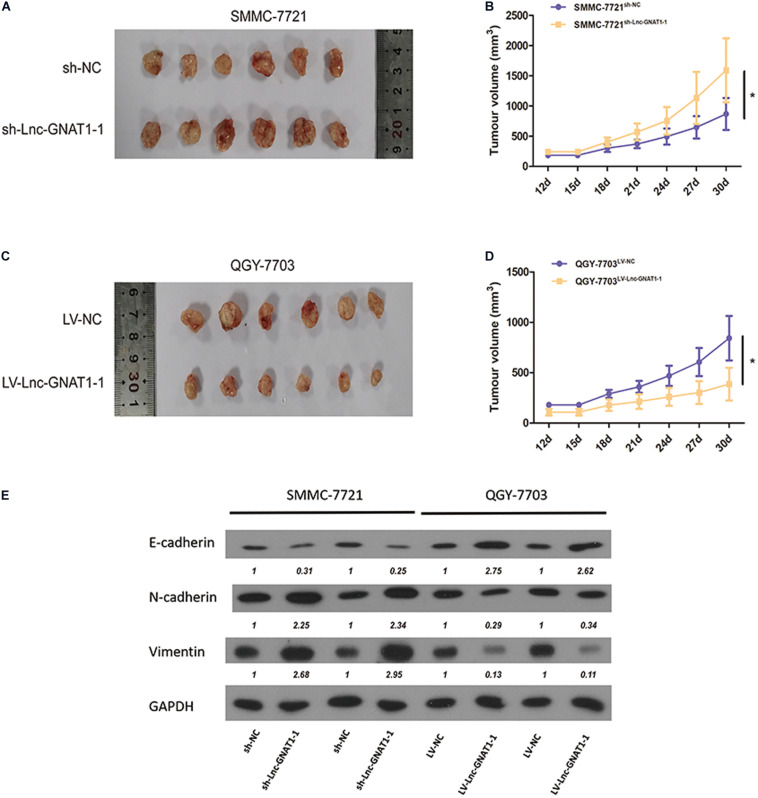
Knockdown of lnc-GNAT1-1 promoted *in vivo* tumor growth of the xenograft model in nude mice, while lnc-GNAT1-1 overexpression exerted the opposite effect. The underlying mechanism may be that lnc-GNAT1-1 downregulated EMT. **(A,B)** Tumor volume changed in mice injected with SMMC-7721 cells after sh-lnc-GNAT1-1 or sh-NC transfection. **(C,D)** Tumor volume changed in mice injected with QGY-7703 cells after LV-lnc-GNAT1-1 or LV-NC transfection. **(E)** The expression levels of E-cadherin, N-cadherin, and vimentin in QGY-7703 cells transfected with LV-lnc-GNAT1-1 or LV-NC and SMMC-7721 cells transfected with sh-lnc-GNAT1-1 or sh-NC. *Significant differences are shown as *P* < 0.05.

SMMC-7721 cells transfected with sh-lnc-GNAT1-1 or sh-NC and QGY-7703 cells transfected with LV-lnc-GNAT1-1 or LV-NC were injected into the subcutaneous area of the necks of nude mice. After dissection, tumor volume was measured, and a greater tumor volume was observed in the sh-lnc-GNAT1-1 group than in the control group (Figures 5A,B). Similarly, reduced tumor volume was found in QGY-7703 cells that had been transfected with LV-lnc-GNAT1-1 (Figures 5C,D).

### Lnc-GNAT1-1 Exerts Regulatory Effects in Liver Cancer Progression by Modulating EMT

The expression levels of EMT-related proteins in SMMC-7721 cells were measured using western blotting assays. Downregulated expression levels of E-cadherin and upregulated expression levels of vimentin and N-cadherin were found in the sh-lnc-GNAT1-1 group (Figure 5E). Upregulated expression levels of E-cadherin and downregulated expression levels of N-cadherin and vimentin were found in QGY-7703 cells with LV-lnc-GNAT1-1 transfection (Figure 5E). These results suggest that lnc-GNAT1-1 inhibited liver cancer progression by downregulating EMT.

## Discussion

In recent years, lncRNAs have attracted increasing attention because of their important regulatory roles in gene expression (Canzio et al., 2019; Lin et al., 2019; Mumbach et al., 2019); an immense amount of investigation has revealed that lncRNAs are involved in cancer onset and development (Esposito et al., 2019; Hu et al., 2019). For example, lncRNA-PVT1 regulates the development of a variety of cancers by interacting with the c-*Myc* gene, acting as a molecular sponge of various microRNAs (miRNAs) and modulating gene transcription and protein expression (Li et al., 2019). LncRNAs can be utilized as diagnostic biomarkers, prognostic biomarkers, and therapeutic targets to improve survival rates (Li et al., 2019). LncRNA-ATB and many other lncRNAs are defined as indispensable cancer-related lncRNAs (Gao and Lian, 2016; Li et al., 2016, 2017); however, the mechanisms and functions of lncRNAs in malignancies remain elusive.

Among these lncRNAs, lnc-GNAT1-1 attracted our attention because of its important effects in the progression of digestive system cancers. Ye et al. (2016) reported that lnc-GNAT1-1 silencing promoted the malignant phenotypes, especially the invasion and migration of colorectal cancer cells, and upregulation of lnc-GNAT1-1 showed the opposite effect. The authors also found that lnc-GNAT1-1 was related to the occurrence of liver metastasis in colorectal cancer, which is rarely reported. The underlying mechanism is thought to be related to the regulation of the RKIP-NF-κB-Snail circuit (Ye et al., 2016). Downregulation of lnc-GNAT1-1 was observed in patients with gastric cancer and lower levels of *Helicobacter pylori* infection and increased expression levels of lnc-GNAT1-1 suppressed gastric cancer growth by inactivating the Wnt/β-catenin pathway (Liu et al., 2018). Against this background, we explored the roles of lnc-GNAT1-1 in liver cancer development and the mechanisms used by lnc-GNAT1-1 to exert modulatory effects on liver cancer progression.

In this present study, we observed obvious downregulation of lnc-GNAT1-1 in liver cancer tissues as well as in cell lines (SMMC-7721, QGY-7703, Huh7, Hep3B, and GSG7701). Among these cell lines, lnc-GNAT1-1 showed higher expression levels in SMMC-7721 and lower levels in QGY-7703. We carried out lnc-GNAT1-1 knockdown in SMMC-7721 cells and overexpressed lnc-GNAT1-1 in QGY-7703 cells. Functional assays showed that downregulation of lnc-GNAT1-1 promoted proliferation, invasion, migration, and *in vivo* growth and reduced cell apoptosis rates in liver cancer cells, while lnc-GNAT1-1 overexpression had the opposite effects.

EMT is crucial for metastasis and invasion of various cancer cells, and upregulation of EMT has been observed in many types of cancer, including liver cancer (Thiery et al., 2009). E-cadherin, N-cadherin, and vimentin are three vital proteins that are normally regarded as EMT markers. E-cadherin inhibits EMT, and vimentin and N-cadherin induce it. The excessive activation of EMT induces loss of cell–cell contacts because of the absence of E-cadherin (Gheldof and Berx, 2013; Wong et al., 2018; Loh et al., 2019). Overexpression of vimentin and N-cadherin contributes to increased cell motility; as a result, these cells easily spread to distant or surrounding tissues (Liu et al., 2015; Loh et al., 2019; Rai and Ahmed, 2019). In liver cancer, studies have found that EMT activation induces liver fibrogenesis and carcinogenesis (Pinzani, 2011) and also promotes the invasion and migration of liver cancer cells (Peng et al., 2018; Zhang et al., 2018). In the present study, the investigation of underlying mechanisms revealed that lnc-GNAT1-1 exerted regulatory effects by modulating the EMT process in liver cancer cells. Our data revealed that lnc-GNAT1-1 silencing increased the expression levels of N-cadherin and vimentin, and reduced E-cadherin expression, while upregulation of lnc-GNAT1-1 showed the opposite result. These findings suggest that upregulation of lnc-GNAT1-1 enhances invasion and migration in liver cancer via the activation of EMT. Normally, lncRNAs exert biological effects in physiological and pathological processes through binding to miRNAs and reducing their levels (Salmena et al., 2011; Sumazin et al., 2011). However, we have not identified relevant miRNAs targeted by lnc-GNAT1-1 to exert anti-tumor effects in liver cancer; this requires further exploration. The associated miRNAs and other potential target genes may be elucidated in our future studies.

## Conclusion

Lnc-GNAT1-1 plays an anti-tumor role in digestive tract cancer development and exerts tumor inhibitory activity to suppress cell proliferation, invasion, and migration and to induce apoptosis in liver cancer cells. We found that lnc-GNAT1-1 inhibited liver cancer progression by regulating EMT. Lnc-GNAT1-1 constitutes a new potential target for improving the quality of life of patients with liver cancer.

## Data Availability Statement

The raw data supporting the conclusions of this article will be made available by the authors, without undue reservation.

## Ethics Statement

The studies involving human participants were reviewed and approved by Affiliated Hospital of Youjiang Medical University for Nationalities. The patients/participants provided their written informed consent to participate in this study. The animal study was reviewed and approved by Affiliated Hospital of Youjiang Medical University for Nationalities.

## Author Contributions

JW and JP designed the study and wrote the manuscript. WW, QT, and LL carried out the experiments. ZL, WL, and YL contributed to data analysis. JP had overall responsibility for the research. All authors approved the final manuscript version.

## Conflict of Interest

The authors declare that the research was conducted in the absence of any commercial or financial relationships that could be construed as a potential conflict of interest.
