# Supplementary material for: Long Non-coding RNA lnc-GNAT1-1 Suppresses Liver Cancer Progression via Modulation of Epithelial–Mesenchymal Transition
Source: Front Genet. 2020 Sep 24;11:1029. doi: 10.3389/fgene.2020.01029 (PMC7541952; doi:10.3389/fgene.2020.01029)
Supplement: Supplementary file 1 [file Presentation_1.PPTX]

## Slide 1
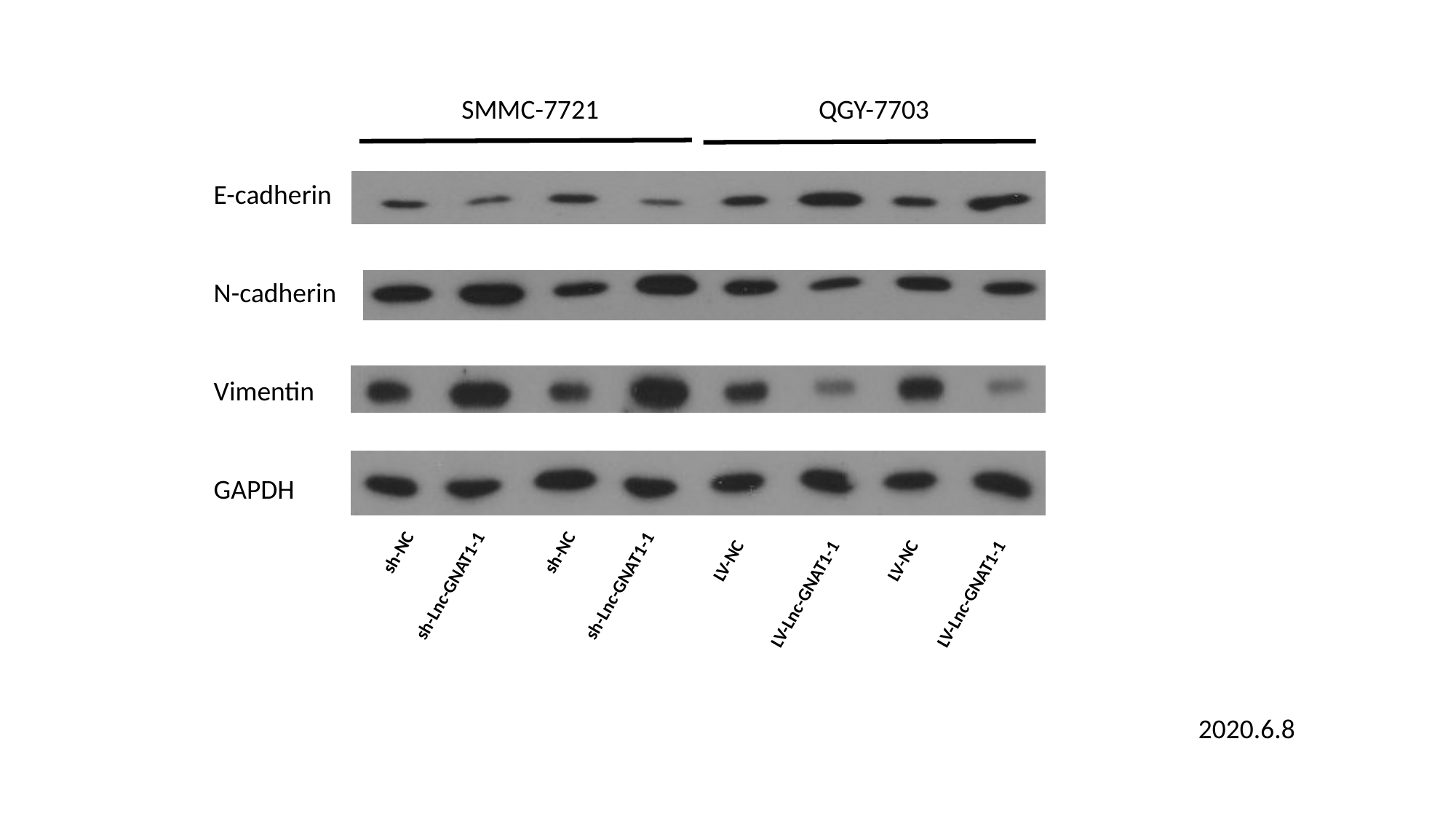

SMMC-7721
QGY-7703
E-cadherin
N-cadherin
Vimentin
GAPDH
sh-NC
sh-NC
LV-NC
LV-NC
sh-Lnc-GNAT1-1
sh-Lnc-GNAT1-1
LV-Lnc-GNAT1-1
LV-Lnc-GNAT1-1
2020.6.8

## Slide 2
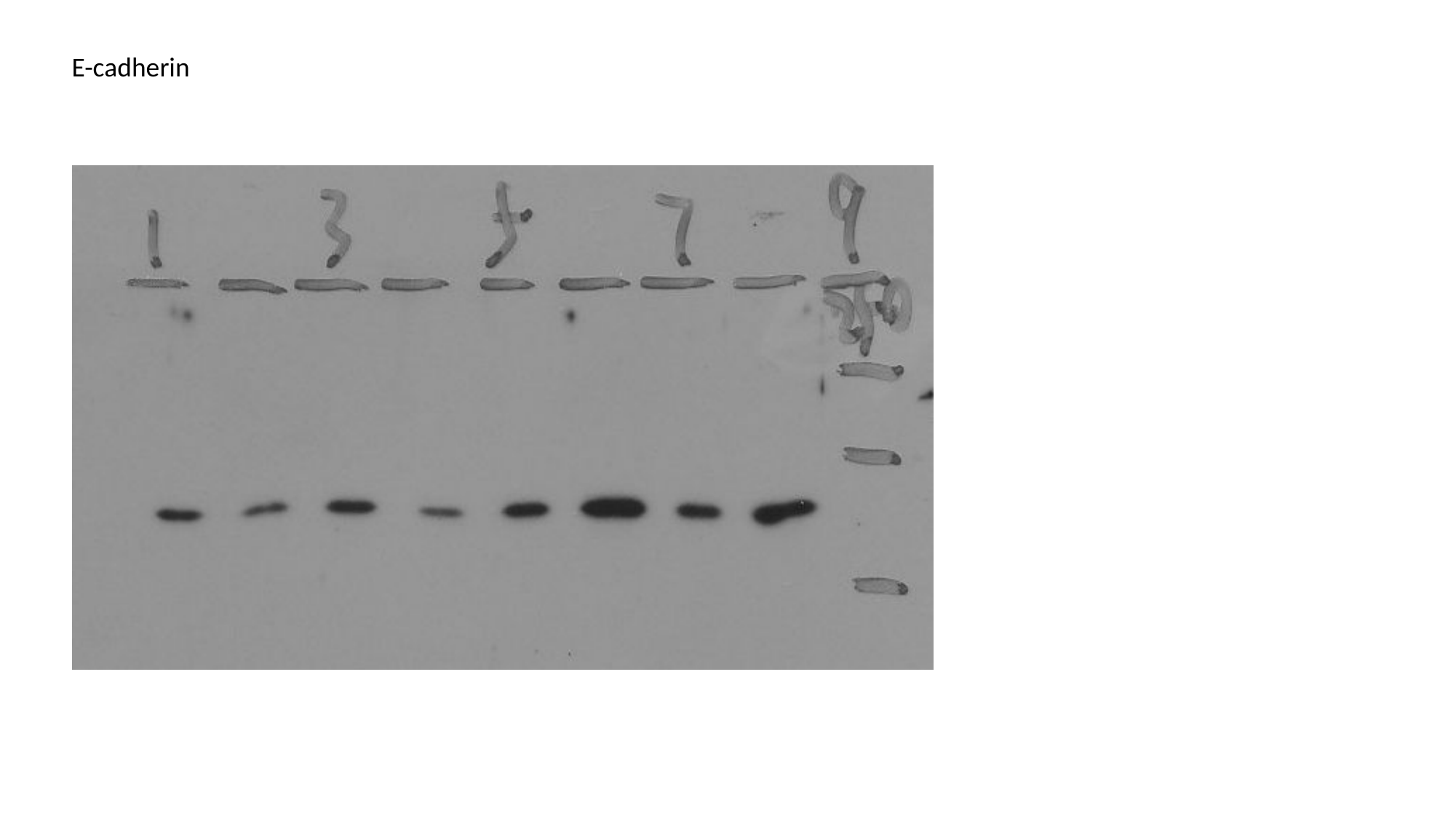

E-cadherin

## Slide 3
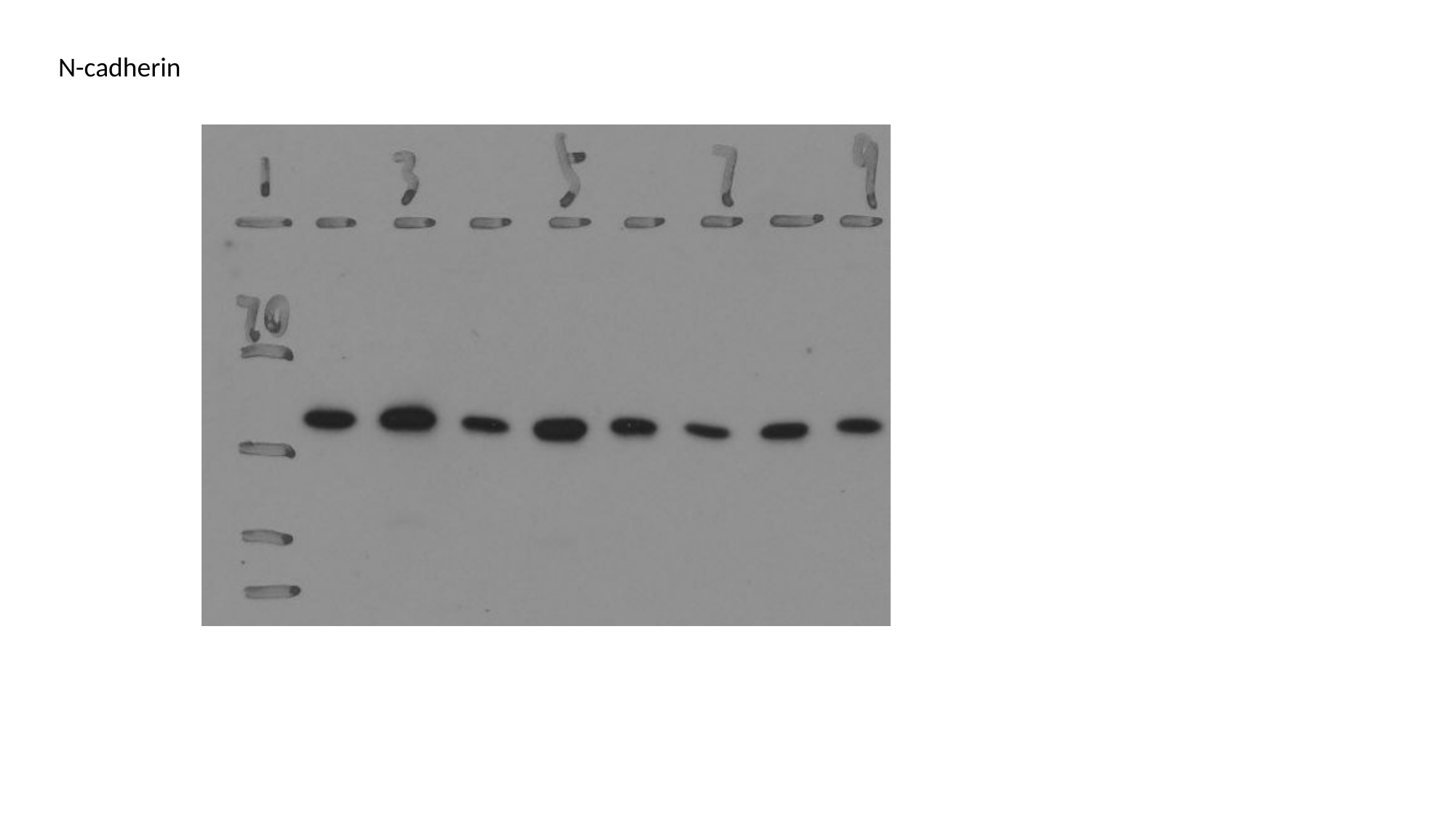

N-cadherin

## Slide 4
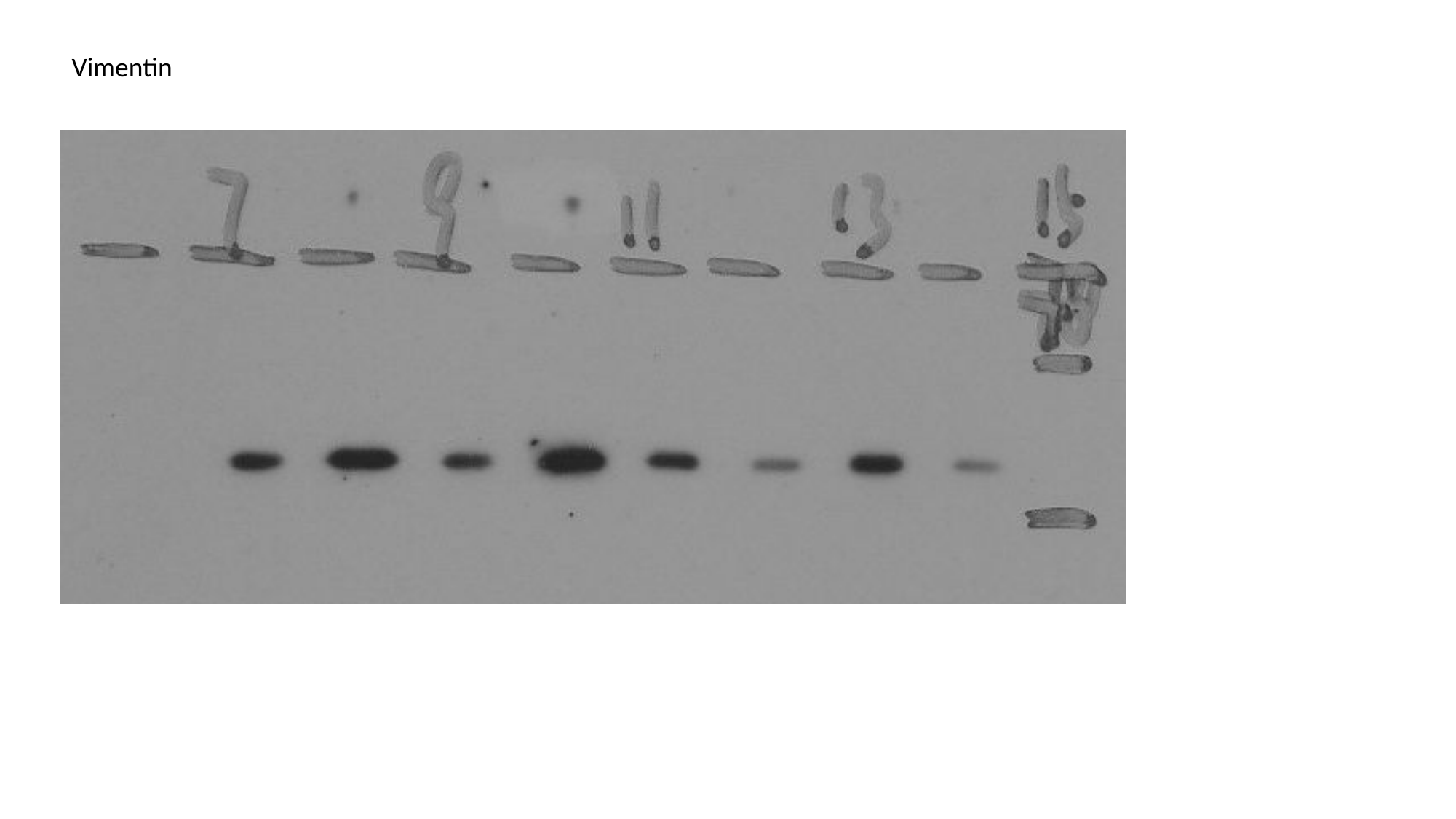

Vimentin

## Slide 5
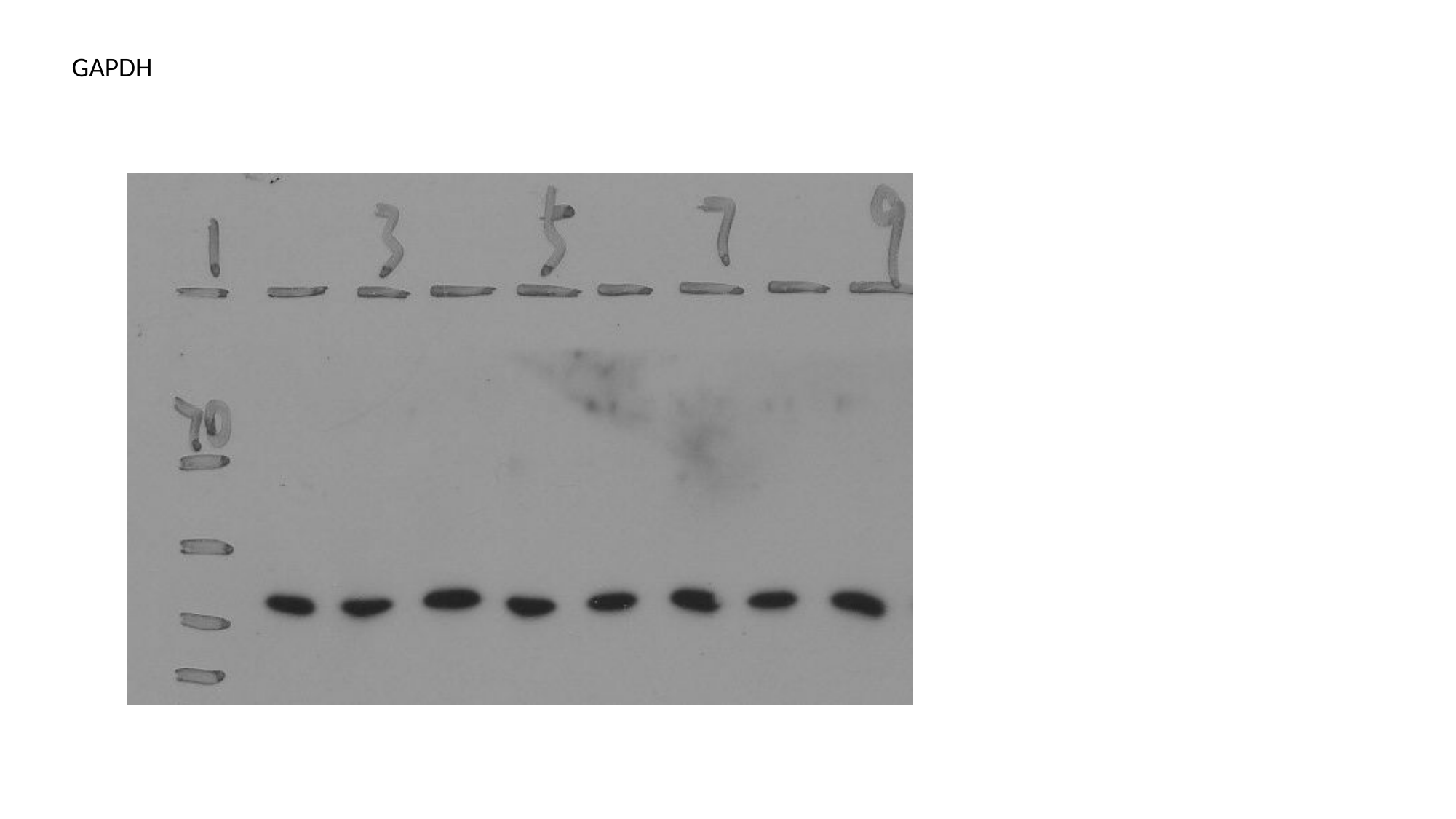

GAPDH
